# Acute Myeloid Leukemia With RUNX1::RUNX1T1 Fusion Transformed From JAK2V617F-Mutated Polycythemia Vera: A Case Report

**DOI:** 10.7759/cureus.92609

**Published:** 2025-09-18

**Authors:** Hidenori Tsukiji, Seiko Miyazaki, Tadafumi Iino, Goichi Yoshimoto, Yasushi Kubota

**Affiliations:** 1 Department of Clinical Laboratory Medicine, Saga-Ken Medical Centre Koseikan, Saga, JPN; 2 Department of Blood Transfusion, Saga-Ken Medical Centre Koseikan, Saga, JPN; 3 Department of Hematology, Saga-Ken Medical Centre Koseikan, Saga, JPN

**Keywords:** acute myeloid leukemia, balanced chromosomal translocation, essential thrombocythemia, jak2v617f mutation, morphology, myeloproliferative neoplasm, polycythemia vera, primary myelofibrosis, runx1::runx1t1

## Abstract

Leukemic transformation is an event that significantly affects the prognosis of patients with Philadelphia chromosome-negative myeloproliferative neoplasms (MPNs). In acute myeloid leukemia (AML) transformed from MPNs, balanced chromosomal translocations, including t(8;21)(q22;q22.1), are extremely rare. A case of secondary AML with *RUNX1*::*RUNX1T1* fusion that evolved from polycythemia vera (PV) is presented. An 87-year-old Japanese man was diagnosed with PV 22 years earlier and had been treated with hydroxyurea and aspirin. Twelve years earlier, a homozygous *JAK2*V617F mutation was identified. Regular blood tests conducted every four months showed leukocytosis (white blood cell count, 14.6 × 10^9^/L), anemia (hemoglobin, 106 g/L), and thrombocytopenia (platelet count, 73 × 10^9^/L). Of the white blood cells, 5.5% were blasts, and the pseudo-Pelger-Huët anomaly and degranulated neutrophils were also observed. Bone marrow aspiration showed 18.2% myeloblasts with varying morphology, characterized by basophilic cytoplasm and wide cytoplasm containing numerous azurophilic granules and perinuclear hofs. The appearance of neutrophils with pink-colored cytoplasm was also noted. Flow cytometry (FCM) showed positivity for myeloid markers, as well as CD34 and CD19, and myeloperoxidase was strongly positive. Morphological findings and FCM results were highly suggestive of AML with the *RUNX1*::*RUNX1T1* fusion gene. Chromosomal analysis identified a translocation t(8;21)(q22;q22.1), and the real-time quantitative polymerase chain reaction detected the *RUNX1*::*RUNX1T1* fusion gene. Based on these findings, the patient was diagnosed with AML with *RUNX1*::*RUNX1T1* fusion that had transformed from PV. In addition, *the JAK2*V617F mutation (homozygous) was detected in peripheral blood at the time of transformation to AML. Leukemic evolution from MPNs often involves morphological changes in addition to genetic and chromosomal abnormalities. Therefore, during the follow-up of MPN, it is important to focus on changes in the blood cell morphology.

## Introduction

Polycythemia vera (PV) is a type of myeloproliferative neoplasm (MPN) that is Philadelphia (Ph) chromosome-negative, along with essential thrombocythemia (ET) and primary myelofibrosis (PMF). Over 95% of cases have a mutation in the *JAK2* gene. PV is characterized by persistent activation of the JAK-STAT signaling pathway, leading to erythrocytosis as the primary feature, accompanied by leukocytosis and thrombocytosis, with an annual incidence of 0.5-4.0 cases per 100,000 persons. The disease typically progresses slowly, but during its course, splenomegaly or thrombosis may develop, and in some cases, it may transform to myelofibrosis (MF) or acute myeloid leukemia (AML) [1,2].

In contrast, AML with the *RUNX1::RUNX1T1* fusion gene has a relatively good prognosis and is frequently observed in de novo AML [3], but transformation from MPN to AML with the *RUNX1*::*RUNX1T1* fusion is extremely rare [4]. Herein, we present an exceptionally rare case of AML with *RUNX1*::*RUNX1T1* fusion that transformed from *JAK2*V617F-mutated PV after a 22-year course, and we discuss the diagnostic clues and clinical implications.

## Case presentation

An 87-year-old Japanese man was diagnosed with PV according to the Polycythemia Vera Study Group criteria in 2002 [5] and started on hydroxyurea therapy. His previous medical history was unremarkable. Phlebotomy was performed several times immediately after PV diagnosis. Subsequently, hematocrit levels remained between 45% and 50% through oral administration of hydroxyurea. He experienced transient ischemic attacks in 2007 and 2011, but no recurrences occurred. In 2012, he was found to be positive for *the JAK2*V617F gene mutation (allele burden was over 80%, homozygous), and aspirin therapy was started. At that time, bone marrow examination showed hyperplasia, with cells at various stages of maturation observed in all three hematopoietic lineages. No increase in blast cells or obvious dysplastic features was noted. These findings confirmed chronic PV with a normal karyotype, and bone marrow biopsy showed mild fibrosis. 

He had been receiving continuous treatment with hydroxyurea. During follow-up every four months, leukocytosis, anemia, and thrombocytopenia developed in 2024 (Table 1). Peripheral blood smears showed 5.5% blasts, along with the pseudo-Pelger-Huët anomaly and degranulated neutrophils. Transformation to acute leukemia was suspected, and bone marrow examination was performed. The bone marrow showed hyperplasia, with cells at all stages of maturation in all three blood cell lines (Table 1).

**Table 1:** Laboratory findings at diagnosis of acute myeloid leukemia transformed from polycythemia vera WBC, white blood cells; RBC, red blood cells; MCV, mean corpuscular volume; MCH, mean corpuscular hemoglobin; MCHC, mean corpuscular hemoglobin concentration; AST, aspartate aminotransferase; ALT, alanine aminotransferase; LDH, lactate dehydrogenase; CRP, C-reactive protein; NCC, nucleated cell count; M/E, myeloid/erythroid

Parameters	Patient Values	Units	Reference Ranges
Complete blood count			
WBC	14.6 × 10^9^	/L	3.3–8.6 × 10^9^
Blast	5.5	%	–
Myelocyte	1.0	%	–
Segmented neutrophils	74.5	%	38.0–74.0
Lymphocytes	9.5	%	16.5–49.5
Monocytes	0.5	%	2.0–10.0
Eosinophils	8.00	%	0.0–8.5
Basophils	1.0	%	0.0–2.5
RBC	2.97 × 10^12^	/L	4.35–5.55× 10^12^
Hemoglobin	106	g/L	137–168
Hemacrit	31.9	%	40.7–50.1
MCV	107.4	fL	83.6–98.2
MCH	35.7	pg	27.5–33.2
MCHC	33.2	g/dL	31.7–35.3
Platelet Count	73 × 10^9^	/L	158–348× 10^9^
Biochemistry			
Total protein	7.0	g/dL	6.6–8.1
Albumin	3.9	g/dL	4.1–5.1
Total Bilirubin	0.4	mg/dL	0.4–1.5
AST	23	U/L	13–30
ALT	28	U/L	10–42
LDH	472	U/L	124–222
Creatine kinase	67	U/L	59–248
Urea nitrogen	20	mg/dL	8.0–20.0
Creatinine	1.28	mg/dL	0.65–1.07
Uric Acid	7.5	mg/dL	3.7–7.8
Sodium	136	mmol/L	138–145
Potassium	5.2	mmol/L	3.6–4.8
Clorine	106	mmol/L	101–108
Calcium	8.8	mg/dL	8.8–10.1
Glucose	119	mg/dL	73–109
CRP	0.13	mg/dL	0.00–0.14
Bone marrow exam			
NCC	30.0 × 10^4^	/μL	10.0–25.0 × 10^4^
Megakaryocytes	7	/μL	50–150
M/E ratio	21.0		1.1–3.5
Neutrophilic series	87.6	%	34.7–78.8
Myeloblast	18.8	%	0.1–0.7
Promyelocytes	4.8	%	1.9–4.7
Myelocytes	4.8	%	8.5–16.9
Metamyelocytes	4.4	%	7.1–24.7
Band neutrophils	4.0	%	9.4–15.4
Segmented neutrophils	28.8	%	3.8–11.0
Eosinophils	13.6	%	1.1–5.2
Basophils	8.4	%	< 0.1
Erythrocytic series	4.4	%	15.0–36.2
Pronormoblasts	0.0	%	0.1–1.1
Basophilic erythroblasts	2.4	%	0.4–2.4
Polychromatophilic erythroblasts	1.4	%	13.1–30.1
Orthochromatic erythroblasts	0.6	%	0.3–3.7
Monocytes	4.8	%	0.0–0.6
Lymphocytes	3.2	%	8.6–23.8

**Figure 1:**
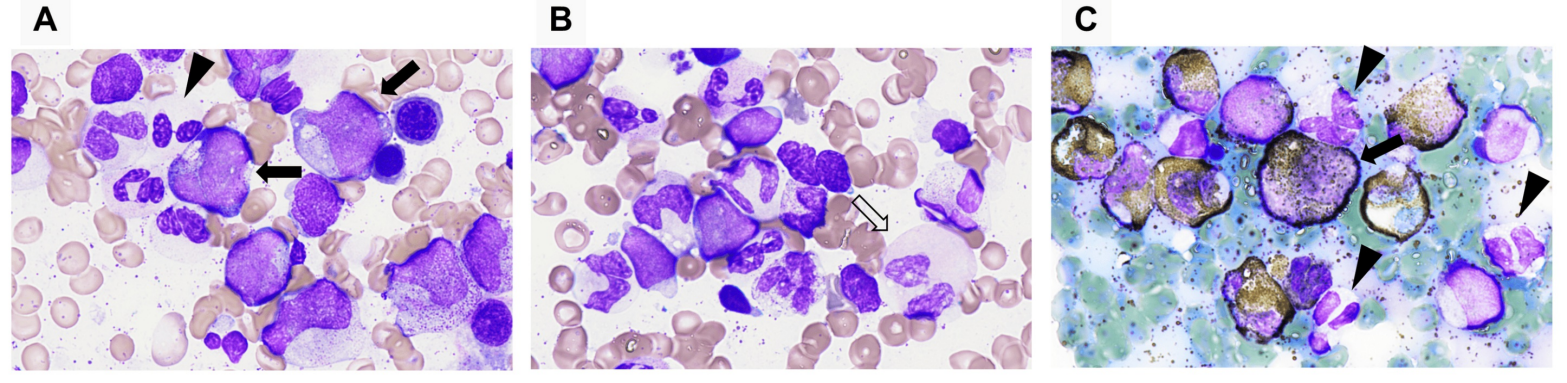
Bone marrow findings. (A) Blast with perinuclear hofs and cytoplasmic granules (closed arrow) and pseudo-Pelger-Huët anomaly and degranulated neutrophils (arrow head) (May-Giemsa staining, ×1,000). (B) Neutrophil with pink-colored cytoplasm (open arrow) (May-Giemsa staining, ×1,000). (C) Myeloperoxidase-positive blasts (closed arrow) and myeloperoxidase-deficient neutrophils (arrow head) (myeloperoxidase staining, ×1,000).

In the granulocyte lineage, similar to the peripheral blood, the pseudo-Pelger-Huët anomaly and hypogranular neutrophils were observed, along with giant neutrophils with pink-colored cytoplasm and myeloperoxidase (MPO)-negative neutrophils. Eosinophils and basophils were also increased. MPO-positive blasts were identified in 18.8% of the cells. The blasts were medium to large in size with variable dimensions, with a nuclear-to-cytoplasmic ratio (N/C ratio) of 70-90%. The cytoplasm contained azurophilic granules and vacuoles. The nuclei exhibited partial clefts or indentations, with fine reticular chromatin and prominent nucleoli (Figure 1).

**Figure 2:**
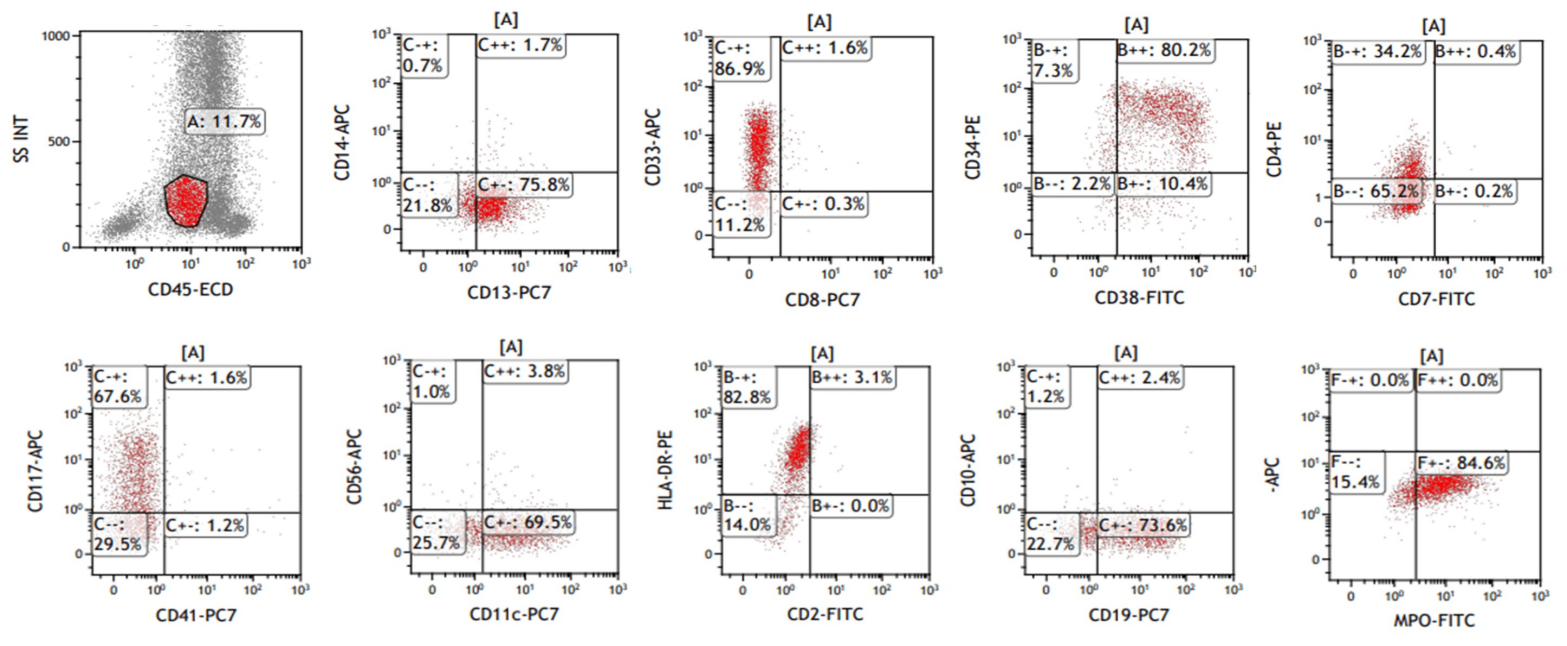
Immunophenotypic findings of flowcytometry analysis. Blasts are positive for CD4, CD11c, CD13, CD19, CD33, CD34, CD38, CD117, HLA-DR, and cytoplasmic MPO.

Flow cytometry (FCM) analysis showed the following phenotypic markers: CD4+, CD11c+, CD13+, CD19+, CD33+, CD34+, CD38+, CD117+, HLA-DR+, and cytoplasmic MPO+ (Figure 2).

**Figure 3:**
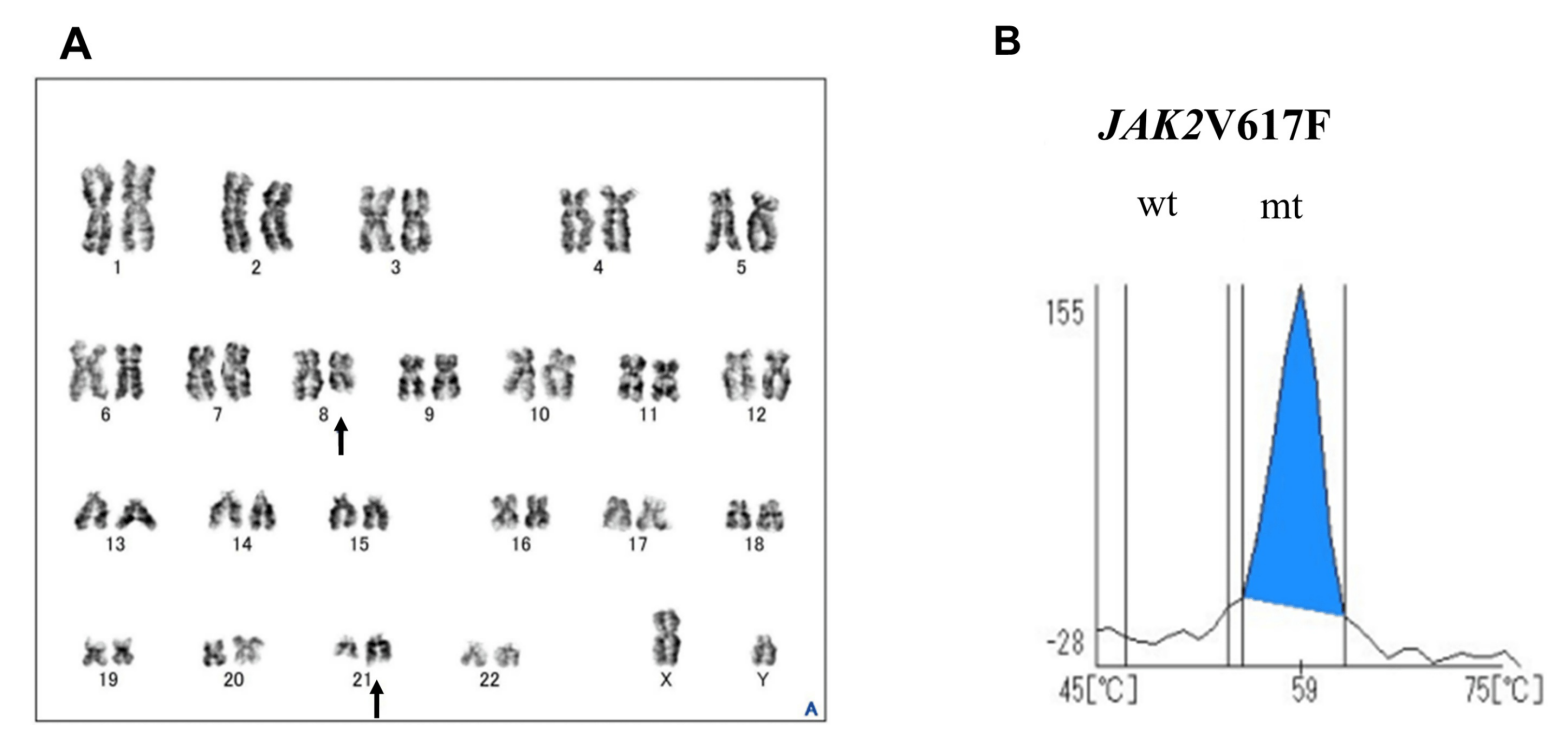
Chromosomal analysis and JAK2V617F mutation analysis. (A) G-banding shows 46,XY,t(8;21)(q22;q22.1) (closed arrow). (B) *JAK2*V617F allele burden shows homozygous (wt, wild type; mt, mutant type). *JAK2*V617F mutation analysis was performed with peripheral blood obtained at the time of leukemia development as the material, using the i-densy IS-5320 (ARKRAY, Inc., Kyoto, Japan), and the melting curve analysis was performed using the genetic analysis support system, MEQNETiDia (ARKRAY, Inc.). The mutation rate of the *JAK2* mutation was analyzed using the *JAK2* mutation analysis software (ARKRAY, Inc.).

The real-time quantitative polymerase chain reaction (RT-qPCR) detected *RUNX1*::*RUNX1T1* at 1.1 × 10^4^ copies/μg RNA. No mutations in the *FLT3* gene were detected. Chromosome analysis showed a karyotype of 46,XY,t(8;21)(q22;q22.1) in 18 of the 20 metaphases examined (Figure 3A). A computed tomography revealed mild splenomegaly. Based on the above findings, the patient was diagnosed with AML harboring the *RUNX1*::*RUNX1T1* fusion gene, which had transformed from PV. *JAK2*V617F mutation analysis was performed in peripheral blood at the time of AML diagnosis using the i-densy IS-5320 (ARKRAY, Inc., Kyoto, Japan) [6] and showed *JAK2*V617F mutation positivity with a homozygous phenotype, and the allele burden was 85% or higher (Figure 3B).

Induction therapy with the combination of venetoclax and azacitidine was administered. Venetoclax was shortened to a 21-day administration. After three courses of treatment, the patient demonstrated treatment resistance, leading to a decision to switch to best supportive care. The patient died approximately eight months after diagnosis of AML with *RUNX1*::*RUNX1T1* fusion.

## Discussion

While leukemic transformation and myelofibrosis are known endpoints of PV, other rare catastrophic complications can occur, such as spontaneous splenic rupture in CML. The present case exemplifies another rare event: transformation to core-binding factor AML. Both scenarios highlight the unpredictable nature of MPNs and the need for vigilance against diverse complications [7]. The overall survival of patients with PV has been reported to range from 13.5 to 24 years. PV is characterized by clinical features such as erythrocytosis, leukocytosis, thrombocytosis, and pruritus. Thrombotic events, MF following PV, and transformation to AML significantly impact the prognosis. The incidence of leukemia 20 years after a PV diagnosis ranges from 7.9% to 17%, with a particularly poor prognosis in cases that progress to leukemia. Risk factors for leukemia development after PV include older age, leukocytosis, venous thrombosis, and abnormal karyotype, among others [8,9]. Genomic profiles of PV and ET that transformed to leukemia indicate that mutations in genes such as *TP53*, *TET2*, *RUNX1*, *ASXL1*, and *EZH2* are frequently associated with this outcome [10]. Regarding the evolution from PV and ET to AML, it is thought that the acquisition of genetic abnormalities leads to the development of leukemia, but the period from diagnosis to leukemic evolution is highly variable. The types of genetic mutations involved in short-term transformation and long-term transformation to AML appear to differ [10].

Interestingly, in de novo AML, balanced chromosomal translocations (*PML*::*RARA*, *RUNX1*::*RUNX1T1*, and *CBFβ*::*MYH11*) are frequently observed, whereas reports of such translocations in AML arising from MPN are scarce [4,11,12]. Furthermore, to the best of our knowledge, there is only one reported case of AML with the *RUNX1*::*RUNX1T1* fusion gene that transformed from PV [13]. In the present case, cytogenetic analysis showed a t(8;21)(q22;q22.1) translocation, and RT-qPCR confirmed the presence of the *RUNX1*::*RUNX1T1* fusion gene. A limitation of our report is the lack of next-generation sequencing data for mutations commonly associated with MPN transformation, such as *TP53*, *ASXL1*, and *SRSF2*. The presence of such additional mutations could provide further insight into the clonal evolution that led to the acquisition of the t(8;21) translocation. Therefore, it remains unclear whether the present case, like previously reported cases, represents a transformation to leukemia triggered by the acquisition of various genetic mutations as a long-term event of PV, or whether it represents an AML development mechanism similar to de novo AML. The patient had been taking hydroxyurea orally for over 20 years. Regarding the association between hydroxyurea and secondary AML in patients receiving long-term hydroxyurea therapy, the use of hydroxyurea alone has been reported to have a limited association with secondary AML [7,14,15]; however, the association between hydroxyurea and the transition of PV to AML with the *RUNX1*::*RUNX1T1* fusion remains unknown.

Cases have been reported in which the *JAK2*V617F mutation disappears with the transformation to leukemia in patients with *JAK2*V617F-positive MPN [16,17]. In contrast, Asou et al. analyzed a case of transformation from ET to AML with *RUNX1*::*RUNX1T1* fusion and found that the *RUNX1*::*RUNX1T1* fusion gene was not detected in peripheral blood leukocytes after chemotherapy, but the *JAK2*V617F mutation was detected in both blasts before chemotherapy and peripheral blood leukocytes after chemotherapy; this suggested that the *JAK2*-mutated ET clones showed leukemic evolution [4]. In the present case, although analysis of *JAK2*V617F in AML cells was not performed, the high *JAK2*V617F allele burden (>85%) in peripheral blood at AML diagnosis strongly suggests that the leukemic blasts with the *RUNX1*::*RUNX1T1* fusion evolved from the original *JAK2*-mutated PV clone, representing a subclone that acquired the t(8;21) translocation.

The morphological features of AML with the *RUNX1*::*RUNX1T1* fusion gene are reported to be lacking in the erythroid and megakaryocytic lineages, but in the granulocytic lineage, characteristic findings include the pseudo-Pelger-Huët anomaly, degranulation, and pink-colored cytoplasm [18]. The blasts are large and vary in size, have a basophilic cytoplasm, and are characterized by numerous azurophilic granules, perinuclear hofs, and Auer rods [19]. In the present case, although Auer rods were not observed, the findings were consistent with those of AML harboring the *RUNX1*::*RUNX1T1* fusion gene in many aspects. Flow cytometry analysis also showed the characteristic immune phenotype of expressing CD34 and CD19, in addition to myeloid markers [20], suggesting the presence of the *RUNX1*::*RUNX1T1* fusion gene.

Hidalgo López et al. reported the bone marrow findings in 58 cases of PV with the blast phase, showing that 88% exhibited myeloid dysplasia, 72% had complex karyotypes, and 55% had *TP53* mutation, with approximately 83% of the total cases diagnosed as AML myelodysplasia-related changes (MRCs) [21]. This finding suggests that, at the time of transformation to leukemia in MPN, including PV, the presence of genetic abnormalities or karyotype abnormalities, along with some form of morphological change, is a sign of PV and other MPNs transforming to leukemia. Therefore, follow-up observation of MPN should include not only blood counts and biochemical tests, but also morphological observations such as peripheral blood smears. Furthermore, given that follow-up in MPN may extend over a long period, as in the present case, capturing morphological changes in blood cells, such as the appearance of blasts or dysplasia, may serve as a helpful tool for understanding the disease process.

## Conclusions

An extremely rare case of AML with the *RUNX1*::*RUNX1T1* fusion transformed from *JAK2*-mutated PV was described in this report. PV progresses relatively slowly, but the prognosis is poor in cases that transform to MF or AML. In the transition phase from MPN to leukemia, detecting not only the appearance of leukemic blasts but also the emergence of morphological dysplasia can serve as a means to grasp the disease progression and provide opportunities for early intervention in treatment. Therefore, in the follow-up of patients with MPN, it is important to pay close attention not only to blood test data, but also to changes in blood cell morphology.
